# Antileishmanial Activity and Synergistic Effects of Amphotericin B Deoxycholate with Allicin and Andrographolide against *Leishmania martiniquensis* In Vitro

**DOI:** 10.3390/pathogens9010049

**Published:** 2020-01-09

**Authors:** Nuchpicha Intakhan, Wetpisit Chanmol, Pradya Somboon, Michelle D. Bates, Vanessa Yardley, Paul A. Bates, Narissara Jariyapan

**Affiliations:** 1 Faculty of Medicine, Graduate PhD Degree Program in Parasitology, Chiang Mai University, Chiang Mai 50200, Thailand; nuchpichaintakhan@gmail.com; 2 Department of Parasitology, Faculty of Medicine, Chiang Mai University, Chiang Mai 50200, Thailand; wetpisitchanmol@gmail.com (W.C.); pradya.somboon@cmu.ac.th (P.S.); 3 Faculty of Health and Medicine, Division of Biomedical and Life Sciences, Lancaster University, Lancaster LA1 4YG, UK; m.bates@lancaster.ac.uk (M.D.B.); p.bates@lancaster.ac.uk (P.A.B.); 4 Faculty of Infectious and Tropical Diseases, London School of Hygiene and Tropical Medicine, London WC1E 7HT, UK; Vanessa.Yardley@lshtm.ac.uk; 5 Department of Parasitology, Faculty of Medicine, Chulalongkorn University, Bangkok 10330, Thailand

**Keywords:** *Leishmaniamartiniquensis*, *Mundinia*, Amphotericin B deoxycholate, allicin, andrographolide, synergistic effect, drug combination

## Abstract

*Leishmania* (*Mundinia*) *martiniquensis* is a causative agent of visceral leishmaniasis, but in HIV-infected patients both visceral and disseminated cutaneous leishmaniasis are presented. Recurrence of the disease after treatment has been reported in some cases indicating that improved chemotherapy is required. In this study, the susceptibility of *L. martiniquensis* to Amphotericin B deoxycholate (AmB), allicin, and andrographolide was evaluated and the synergistic effects of allicin or andrographolide combined with AmB against *L. martiniquensis* intracellular amastigotes in mouse peritoneal exudate macrophages (PEMs) were investigated in vitro for the first time. The results showed that *L. martiniquensis* was highly susceptible to AmB as expected, but allicin and andrographolide had selectivity index (SI) values greater than 10, indicating promise in both compounds for treatment of host cells infected with *L. martiniquensis*. Four AmB/allicin combinations presented combination index (CI) values less than 1 (0.58–0.68) for intracellular amastigotes indicating synergistic effects. The combination with the highest dose reduction index (DRI) allowed an approximately four-fold reduction of AmB use in that combination. No synergistic effects were observed in AmB/andrographolide combinations. The data provided in this study leads for further study to develop novel therapeutic agents and improve the treatment outcome for leishmaniasis caused by this *Leishmania* species.

## 1. Introduction

Leishmaniasis is an emerging disease in Thailand and South East Asia, in which most of the human cases to date have presented with the clinical features of disseminated and/or visceral leishmaniasis accompanied by HIV infection [1,2]. The number of clinically and parasitologically confirmed cases remains relatively small (about 25), however, the appearance of leishmaniasis in South East Asia has raised important concerns for two reasons. The first concern is that clinical disease may become more widely established in the region than appears to be the case at present. For example, in the adjacent region of South Asia visceral leishmaniasis is a major public health challenge [3]. The second concern is the suspicion that there is already a much higher underlying rate of infection than current numbers suggest, for example, in a recent study a prevalence of 25.1% was indicated in HIV patients in southern Thailand [4].

*Leishmania* (*Mundinia*) *martiniquensis* is the most frequent cause of leishmaniasis in Thailand [1,2]. The parasite was first isolated in 1995 on the island of Martinique [5] and fully described in 2014 [6]. This new species has been placed in a new subgenus *L.* (*Mundinia*) [7] and studied relatively little. In Thailand, Pothirat et al. (2014) reported the first case of autochthonous visceral leishmaniasis in northern Thailand and the aetiological agent was identified as *L. martiniquensis* [8]. In South East Asia *L. martiniquensis* can present in a range of clinical presentations, most frequently as visceral leishmaniasis in patients with no known underlying immunodeficiency [8]. However, when accompanied by HIV-infection, both visceral and/or disseminated cutaneous leishmaniasis has been reported [9], similar to elsewhere [10].

Amphotericin B deoxycholate (AmB), a sterol-complexing agent, is the only first-line drug currently available in Thailand and has been used to treat most of the cases [1]. However, the susceptibility of *L. martiniquensis* to AmB has not been previously investigated and recurrence of the disease after treatment has occurred in some cases, in both seronegative and immunocompromised patients including HIV-infected patients, indicating that improved chemotherapy is required [1,11]. Disadvantages of current therapy with AmB include low solubility leading to poor bioavailability, renal toxicity, other occasional serious side effects, the need for administration by slow infusion, and infusion-associated reactions such as thrombophlebitis, chills, and high fever with rigor [12]. Moreover, the requirement for long periods of parenteral administration, frequently requiring hospitalization, has limited the clinical use of AmB [13]. Unfortunately, the less toxic liposomal formulation of AmB, Ambisome^®^, is not available in Thailand, and, therefore, new drugs or more effective combinations are required. The use of combinations of different drugs and/or compounds may also bring significant advantages and better therapeutic effects than each of the substances alone.

Allicin and andrographolide are readily available natural products that have shown promise as antileishmanial agents. Allicin has been reported to be effective against the intracellular stages of *L. donovani* and *L. infantum* without substantial cytotoxicity for mammalian cells [14,15], and against the in vitro growth of *L. mexicana* and *L. infantum* promastigotes [16]. Allicin works with AmB against intracellular amastigotes of *L. donovani* and *L. infantum* with a moderate synergistic effect with a two-fold reduction of AmB [15]. It has also shown inhibition of the growth of *L. major* promastigotes [17]. In addition, Corral et al. (2016) have reported that allicin causes necrotic death in *L. infantum* [17]. Another interesting compound, andrographolide, has shown strong general antileishmanial effects against *L. donovani*-infected macrophages in vivo [18]. It has also been reported to have antiplasmodial activity against *Plasmodium falciparum* erythrocytic stages [19] and antitrypanosomal activity against *Trypanosoma brucei* [20]. Neither allicin nor andrographolide have been tested for their effects on *L. martiniquensis*.

The aims of this study were to evaluate the susceptibility of *L. martiniquensis* to AmB, allicin, and andrographolide, followed by the investigation of any synergistic effects of allicin or andrographolide combined with AmB against *L. martiniquensis* intracellular amastigotes in mouse peritoneal exudate macrophages (PEMs). The results provided by this study give crucial baseline information on the efficacy of the current first-line treatment for leishmaniasis in Thailand and South East Asia, and also enable assessment of potential improvements to the treatment regime using a combination chemotherapy approach.

## 2. Results

### 2.1. Antileishmanial Activity against Promastigotes

Exposure of *L. martiniquensis* promastigotes to AmB, allicin, and andrographolide demonstrated that the drug and both compounds were able to inhibit parasite growth. The half maximal inhibitory concentration (IC_50_) values of allicin and andrographolide were 7.70 (7.69–7.71) μg/mL and 4.04 (4.03–4.05) μg/mL, respectively. The IC_50_ value of AmB was 0.040 (0.039–0.041) μg/mL, showing the *L. martiniquensis* promastigotes were much more sensitive to AmB than either allicin or andrographolide alone.

### 2.2. Cytotoxicity on BALB/c Peritoneal Macrophages

The cytotoxicity of AmB, allicin, and andrographolide on mouse PEMs was determined because these are mammalian cells, but also because they were used as the host cells for *L. martiniquensis* infection assays. The 50% cytotoxic concentration (CC_50_) values for AmB, allicin, and andrographolide were 54.0 (47.2–60.8), 13.9 (13.4–14.4), and 6.60 (6.03–7.17) μg/mL, respectively. These results show that andrographolide was the most toxic of the three, although overall there were not large differences in their effects on PEMs.

### 2.3. Antileishmanial Activity against Intracellular Amastigotes

**Figure 1:**
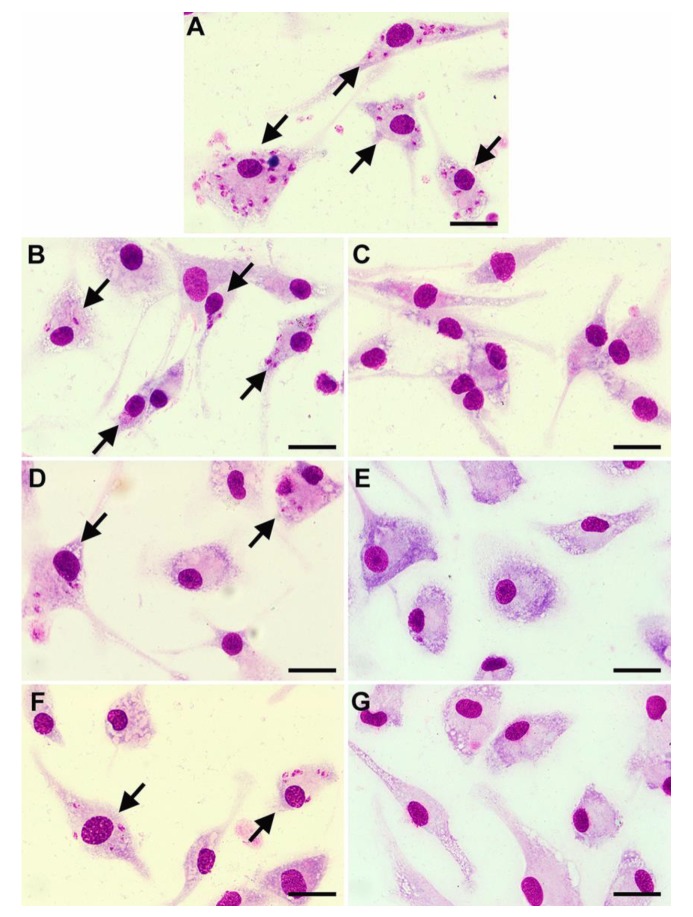
Photomicrographs showing representative images of *L. martiniquensis*-infected macrophages from control and treated groups stained with Giemsa. Untreated control (**A**) *Leishmania*-infected macrophages treated with 0.02 μg/mL of Amphotericin B deoxycholate (AmB), (**B**) *Leishmania*-infected macrophages treated with 0.63 μg/mL of AmB, (**C**) *Leishmania*-infected macrophages treated with 0.63 μg/mL of allicin, (**D**) *Leishmania*-infected macrophages treated with 10 μg/mL of allicin, (**E**) *Leishmania*-infected macrophages treated with 0.31 μg/mL of andrographolide, (**F**) *Leishmania*-infected macrophages treated with 10 μg/mL of andrographolide (**G**). Arrows indicate infected macrophages. Bar: 50 μm.

Although the efficacy of potential drugs against *Leishmania* promastigotes is useful information, as an intracellular parasite it is essential that any drug is able to access the amastigote forms of the parasite inside their host cells, as well as displaying selective toxicity for the parasites within. The activity of compounds against amastigote forms of *L. martiniquensis* was determined in *Leishmania*-infected macrophages. The microscopic observations of Giemsa-stained cells demonstrated that the drug and both compounds had effects on intracellular parasites. Untreated control showed numerous amastigotes in macrophages (Figure 1A). AmB reduced the number of intracellular amastigotes at 0.02 μg/mL (Figure 1B), and macrophages were free of parasites after treatment with AmB at 0.63 μg/mL (Figure 1C). Allicin was less effective than AmB but was able to reduce intracellular amastigotes at 0.63 μg/mL and no intracellular amastigotes were found after treatment with 10 μg/mL of allicin (Figure 1D,E). Similarly, andrographolide was able to reduce intracellular amastigotes at 0.31 μg/mL and no parasites were observed after treatment with 10 μg/mL of andrographolide (Figure 1F,G). AmB drug and both compounds reduced the infection index in a dose-dependent manner. The IC_50_ values for allicin and andrographolide were 0.59 (0.49–0.69) μg/mL and 0.45 (0.35–0.55) μg/mL, respectively, whereas the IC_50_ value of AmB was 0.0152 (0.0147–0.0157) μg/mL. These results are in broad agreement with those obtained with promastigotes. To compare their selective toxicity, the selectivity index (SI) values (CC_50_/IC_50_) were calculated. The SI values were 23.55, 14.66, and 3553 for allicin, andrographolide, and AmB, respectively, confirming that AmB was the most effective of the three compounds.

### 2.4. Activity of Synergistic Combinations against Intracellular Amastigotes

**Figure 2:**
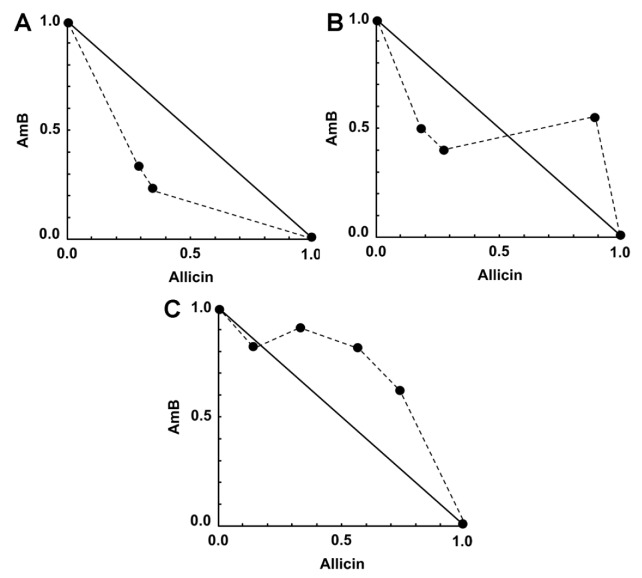
Representative normalized isobolograms of the interaction of AmB/allicin on *L. martiniquensis* intracellular amastigotes. (**A**) 0.0025 μg/mL AmB plus 0.16 or 0.32 μg/mL allicin. (**B**) 0.005 μg/mL AmB plus 0.16, 0.32, or 0.64 μg/mL allicin. (**C**) 0.01 μg/mL AmB plus 0.16, 0.32, 0.64, or 1.28 μg/mL allicin. Data points (dots) located below, on, or above the line indicate synergy, additivity, or antagonism, respectively.

The possible synergistic effects of allicin or andrographolide when combined with AmB against intracellular amastigotes was investigated by using the Chou-Talalay combination index method. Allicin and andrographolide at 0.64 μg/mL and AmB at 0.01 μg/mL provided an approximate 50% growth inhibition, which were similar to previous IC_50_ results. The percentage of infected macrophages after 48 h of incubation with no treatment (untreated control) was in a range of 50–60% (data not shown). The percentage of growth inhibition (compared with untreated control) obtained from the checkerboard method was used to determine combination index (CI) value of each combination in order to identify the type of interaction (synergism, addition, or antagonism). An interaction between AmB and allicin was found from synergism with the lowest concentration of AmB used (0.0025 μg/mL) plus allicin (0.16 μg/mL) to moderate antagonism with the highest concentration of AmB used (0.01 μg/mL) plus allicin (0.64 μg/mL). However, four combinations of AmB/allicin (0.0025:0.16, 0.0025:0.32, 0.005:0.16, and 0.005:0.32 μg/mL) were classified as synergism and one combination (0.01:0.16 μg/mL) was classified as nearly additive (Table 1). Similarly, a graphical representation (isobologram) representing AmB and allicin interactions indicates synergy for four combinations by showing four data points below the line of additivity (Figure 2). Combinations of AmB/allicin with synergistic effects allowed dose reductions for a given effect level. The combination of AmB 0.0025 μg/mL plus allicin 0.32 μg/mL showed the highest dose reduction index (DRI) with approximately a four-fold reduction of AmB use, as shown in Table 1. No cytotoxicity assay on macrophages was performed for those combinations with synergism effect as the combinations used lower concentration of drug/compound than the drug or compound alone and no host cell damage was observed.

**Figure 3:**
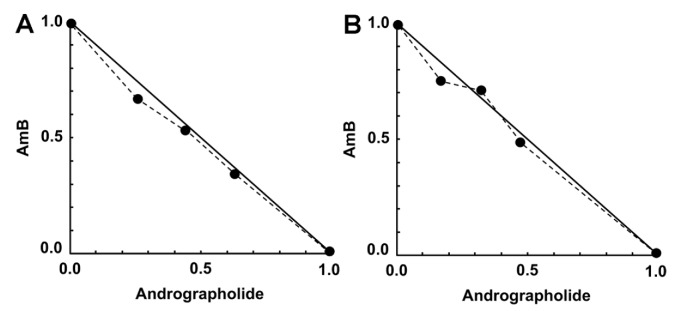
Representative isobolograms of the interaction of AmB/andrographolide on *L. martiniquensis* intracellular amastigotes. (**A**) 0.0025 μg/mL AmB plus 0.08, 0.16, or 0.32 μg/mL andrographolide. (**B**) 0.005 μg/mL AmB plus 0.08, 0.16, or 0.32 μg/mL andrographolide. Data points (dots) located on the line indicate additivity.

An interaction of AmB and andrographolide was found from nearly additive to antagonism (Table 2). Six out of twelve combinations of AmB/andrographolide showed additive effects on intracellular parasites. The isobolograms for the interaction between AmB and andrographolide demonstrate six data points located on the line of additivity indicating nearly additive effects (Figure 3).

## 3. Discussion

The action of AmB against *L. martiniquensis* was investigated in both promastigote and intracellular amastigote assays, with IC_50_ values comparable to those seen with other *Leishmania* species [21,22]. Importantly, this confirms the logical use of AmB for chemotherapy of *L. martiniquensis* infection. Although these parasites are found within a distinct subgenus, *Leishmania* (*Mundinia*), and are therefore genetically distinct from other species of *Leishmania*, they must be sufficiently similar in terms of their sterol composition for AmB to be an effective drug. With the goal of improving therapeutic applications for *L. martiniquensis* infection by reduction of the undesirable side effects of AmB, we then evaluated the susceptibility of *L. martiniquensis* to allicin and andrographolide, followed by investigation of the synergistic effects of allicin or andrographolide combined with AmB against intracellular amastigotes in PEMs. In the present study, allicin and andrographolide were able to act directly to inhibit the growth of extracellular promastigotes of *L. martiniquensis*. Those antileishmanial activities were also effective against intracellular amastigotes of *L. martiniquensis* in PEMs by showing SI values greater than 10, which present promising results for the use of allicin and andrographolide for treatment of host cells infected with *L. martiniquensis* [23,24].

To determine whether either compound was able to augment AmB monotherapy against intracellular amastigotes in PEMs, combinations of allicin or andrographolide with AmB were investigated for their efficacy. Our results showed that four AmB/allicin combinations demonstrated synergistic effects, with the combination of 0.0025 μg/mL of AmB plus 0.32 μg/mL of allicin producing the highest DRI, about a four-fold reduction of AmB used in the combination. Allicin has also been reported to act synergistically with AmB against intracellular amastigotes of *L. donovani* and *L. infantum.* The interaction of allicin and AmB against these *Leishmania* species was a moderate synergistic effect with a two-fold reduction of AmB [15]. The combination of allicin and AmB was also effective in other organisms, for example, An et al. (2009) found that allicin was able to enhance the oxidative damage activity of AmB to destroy *C. albicans* [25]. Regarding mode of action in *L. infantum* promastigotes, allicin alone directly interfered with calcium homeostasis and induced oxidative stress leading to mitochondrial dysfunction [26]. Furthermore, previous studies have reported that allicin reacted with thiols that caused a defect of trypanothione reductase in defense against reactive oxygen species (ROS) [27,28]. Clearly, in our study allicin was able to diffuse and permeate across cell membranes into macrophages, but the mechanism of allicin on intracellular amastigotes remains unknown [29,30]. AmB effects *Leishmania* parasites by binding to ergosta-type sterols, these being constituents of the plasma membrane, and then forming pores [31,32]. These results in leakage of potassium ions leading to toxic effects through the absence of intracellular ionic substances, and also induces ROS-based oxidative damage. Based on our findings, the synergistic effect of allicin with AmB against intracellular amastigotes of *L. martiniquensis*, might be explained by enhancing the ability of AmB to disrupt membrane function, as well as potential direct effects of allicin on trypanothione reductase or mitochondrial function.

In the case of AmB and andrographolide combinations, no synergistic effect was observed in any combination. Further, the AmB and andrographolide combinations were at best additive, but in many cases showed strong antagonistic effects against intracellular amastigotes of *L. martiniquensis*. In cancer cell therapy, andrographolide was able to work synergistically with anticancer agents to inhibit the tumor growth by arresting cell cycle and inducing cell apoptosis [33,34,35,36,37]. So far, there are no reports of synergistic effects of andrographolide in combination with AmB against any other *Leishmania* species. In our study, andrographolide was able to inhibit growth of *L. martiniquensis* promastigotes and affect intracellular amastigotes. Recently, nanoparticle formulations of andrographolide have been used to target and treat *L. donovani* infected macrophages [38]. Furthermore, the successful treatment of *L. donovani*-infected hamsters was achieved using the encapsulated andrographolide in mannose-grafted liposomes [18]. Therefore, using carrier systems or new formulations might improve the efficacy of AmB and andrographolide combinations. However, assessment of the effects of the combinations in this present study should also be performed using an in vivo experimental model for *L. martiniquensis*, as some combinations that were effective in vitro might still act as useful therapeutic partners.

In conclusion, in this study the susceptibility of *L. martiniquensis* to AmB, allicin, and andrographolide was evaluated and the synergistic effects of allicin or andrographolide combined with AmB against *L. martiniquensis* intracellular amastigotes in PEMs were investigated in vitro for the first time. *L. martiniquensis* was susceptible to both allicin and andrographolide. However, only allicin worked synergistically with AmB and reduced drug use in the combination against intracellular amastigotes. These results might lead to further studies to improve the therapeutic outcome for the *Leishmania* species.

## 4. Materials and Methods

### 4.1. Ethics Statement

All the protocols used for the care and use of laboratory animals were reviewed and approved by the Ethics Committee on Animal Use of the Laboratory Animal Center, Chiang Mai University (Protocol number 2561/MC-0008).

### 4.2. Parasites and Culture

*L. martiniquensis* (MHOM/TH/2013/LSCM3) originally isolated from a bone marrow sample of a disseminated leishmaniasis patient [39] was used this study. For routine culture, promastigotes were cultured in M199 medium (GE Healthcare Life Sciences, Logan, UT, USA), which was supplemented with 10% (*v*/*v*) heat-inactivated fetal bovine serum (FBS), 1% basal medium eagle vitamins (BME) (Sigma-Aldrich, St. Louis, MO, USA), and 25 μg/mL gentamicin sulfate (Sigma-Aldrich, St. Louis, MO, USA), pH 6.8 at 26 °C.

### 4.3. Drug and Compounds

Amphotericin B deoxycholate (250 μg/mL) was purchased from Gibco (Grand Island, NY, USA). Allicin (liquid Allisure, 1000 ppm) was purchased from Allicin International Ltd. (Rye, East Sussex, UK). Andrographolide (100 mg) was purchased from Sigma (St. Louis, MO, USA). A stock solution of andrographolide (500 μg/mL) was prepared in dimethylsulfoxide (DMSO) and stored at −20 °C. The final DMSO concentration (0.4%) had no effect on parasite growth in controls. Dilutions of AmB, allicin, and andrographolide were prepared in the culture medium on the day of treatment and immediately used.

### 4.4. Promastigote Assay

The antileishmanial activity of compounds against promastigotes was determined using alamarBlue™ Cell Viability Reagent (Thermo Fisher Scientific, Waltham, MA, USA) as described by the manufacturer with some modifications. Exponential phase promastigotes of *L. martiniquensis* (2 × 10^6^ cells/mL) prepared in M199 medium (supplemented with 10% FBS, 1% BME vitamins, and 25 μg/mL gentamicin sulfate) were plated in 96-well culture plates (Nunc, Roskilde, Denmark) (50 µL/well). The promastigotes then received 50 µL of medium alone (control) or containing different concentrations of AmB (0.0025–0.32 μg/mL), allicin (0.63–40 μg/mL), or andrographolide (0.14–17.5 μg/mL), to obtain a final volume of 100 µL, and incubated at 26 °C. After exposure to drug and compounds for 48 h, 10 μL of alamarBlue™ Cell Viability Reagent was added to each well, and incubation continued for 24 h at 26 °C. Promastigote proliferation was determined using a plate reader (Synergy H4 Hybrid Microplate reader, BioTek, Winooski, VT, USA) at wavelengths of 570 and 600 nm. Wells without cells and the maximal concentration from each drug or compound and wells with culture medium and alamarBlue™ Cell Viability Reagent (10% *v*/*v*) were included as controls. The IC_50_ value, defined as the concentration of drug or compound required to inhibit 50% promastigote growth, was determined from a sigmoidal dose response curve generated using Graphpad prism 6 software (Graphpad software Inc., San Diego, CA, USA). These assays were performed in three independent experiments and in triplicate within each experiment. Results are expressed as mean 95% confidence interval.

### 4.5. Cytotoxicity Assay

The use of mouse-derived PEMs can be advocated as an efficient, reliable, relatively quick, and cost-effective tool for evaluation of antileishmanial drug or compound efficacy in vitro [40]. In this study, PEMs were collected from female 8- to 12-week-old BALB/c mice (purchased from Nomura Siam International Co., Ltd., Bangkok, Thailand) using the method described by Zhang et al. [41]. Trypan blue (Sigma-Aldrich, St. Louis, MO, USA) was used to test for the initial viability of PEMs. The PEMs were suspended in RPMI-1640 medium (GE Healthcare Life Sciences, Logan, UT, USA) (supplemented with 10% FBS, 25 μg/mL gentamicin sulfate) and plated in 96-well culture plates (2.5 × 10^4^ viable cells/well; 100 µL/well) and incubated for 24 h at 37 °C, 5% CO_2_ to allow cell adherence. After that the PEMs were incubated with their respective medium alone (control) or containing AmB, allicin, or andrographolide at different concentrations (1.4–180 μg/mL) for 72 h at 37 °C, 5% CO_2_. Viability of cells in presence of AmB, allicin, or andrographolide was determined using the alamarBlue™ assay. Briefly, after 72 h of incubation, 10 μL of alamarBlue™ Cell Viability Reagent was added to each well and incubation continued for 4 h at 37 °C, 5% CO_2_ before reading absorbance. Cell viability was determined using a plate reader at wavelengths of 570 and 600 nm. Wells without cells and the maximal concentration from each drug or compound and wells with culture medium and alamarBlue™ Cell Viability Reagent (10% *v*/*v*) were included as controls. The CC_50_ value, defined as the concentration of drug or compound required to induce 50% cell death, was determined from a sigmoidal dose response curve using Graphpad prism 6 software (Graphpad Solfware Inc., San Diego, CA, USA). These assays were performed in three independent experiments and in triplicate within each experiment. Results are expressed as mean 95% confidence interval.

### 4.6. Preparation of Promastigotes to Infect Murine Macrophages

The preparation of promastigotes for infection in murine macrophages was performed as follows. *L. martiniquensis* parasites derived from infected BALB/c mice (sixteen weeks post infection) were used. The spleen of an infected mouse was removed aseptically and briefly placed in sterile phosphate buffer saline. The spleen tissues were then minced in Schneider’s insect medium (SIM) (Sigma-Aldrich, St. Louis, MO, USA) supplemented with 10% FBS and 25 μg/mL gentamicin sulfate and strained using a cell strainer (SPL Life Sciences Co., Ltd., Gyeonggi-do, Korea), using aseptic techniques. The resulting opaque suspension was transferred to a 50 mL centrifuge tube (Nunc, Roskilde, Denmark), topped up to 50 mL with SIM supplemented with 10% FBS, 25 μg/mL gentamicin sulfate, and centrifuged at 26 °C, 1500× *g* for 10 min. The supernatant medium was discarded. The pellet was resuspended in SIM supplemented with 10% FBS, 25 μg/mL gentamicin sulfate, and incubated for 3 days at 26 °C to allow promastigotes to grow. Such promastigotes were then subpassaged into RPMI-1640 medium supplemented with 20% FBS, pH 5.5, 25 μg/mL gentamicin sulfate to stimulate growth followed by metacyclogenesis [42] and incubated for 5 days at 26 °C. Stationary phase promastigote cultures with >70% metacyclic promastigotes were used to infect PEMs.

### 4.7. Intracellular Amastigote Assay

To evaluate effect of drug or compounds alone on intracellular amastigotes of *L. martiniquensis*, *Leishmania*-infected murine macrophages were prepared as follows. PEMs were collected from BALB/c mice as described above, then suspended in RPMI-1640 medium supplemented with 10% FBS, 25 μg/mL gentamicin sulfate and plated in 8-well chamber slides (Nunc, Roskilde, Denmark) (5 × 10^4^ viable cells/well; 200 µL/well). Cultures were incubated for 24 h at 37 °C, 5% CO_2_ to allow cell adherence. Adherent cells were infected with stationary phase promastigotes of *L. martiniquensis* in RPMI-1640 medium supplemented with 2% FBS, 25 μg/mL gentamicin sulfate at a parasite/macrophage ratio of 10:1 to achieve an optimal level of infection (approximate 90% infection) before starting drug sensitivity assay. Infected cells were washed to remove non-internalized promastigotes and the medium replaced with RPMI-1640 medium supplemented with 2% FBS, 25 μg/mL gentamicin sulfate containing different concentrations of AmB (0.002–0.63 μg/mL), allicin (0.04–10 μg/mL), and andrographolide (0.04–10 μg/mL) and incubated for 48 h at 37 °C, 5% CO_2_. After the treatment, infected macrophages were fixed with absolute methanol and stained with 5% Giemsa’s stain solution. Intracellular amastigotes and infected macrophages were counted microscopically (from at least 300 macrophages). The infection index was calculated by multiplying the percentage of infected macrophages by the average number of intracellular amastigotes per infected cells [43]. The IC_50_ value was determined from a sigmoidal dose response curve using Graphpad prism 6 software. Results are expressed as mean 95% confidence interval of at least three independent experiments, each performed in triplicate. The SI of the drug or compounds was calculated from the ratio of the CC_50_ for macrophages and the IC_50_ for parasites.

### 4.8. Drug Combination Assay on Intracellular Amastigotes

Combination effects of drug and compounds against intracellular amastigotes were determined by using checkerboard assays. AmB, allicin, and andrographolide at concentrations near or equal to their IC_50_ values were prepared in double concentration and serially two-fold diluted in RPMI-1640 medium supplemented with 2% FBS, 25 μg/mL gentamicin sulfate. Each compound with different dilutions was combined with different dilutions of the drug. The matrix yielded 9 different combinations for AmB/allicin and 12 different combinations for AmB/andrographolide. *L. martiniquensis*-infected murine macrophages were prepared in 8-well chamber slides with a parasite/macrophage ratio of 10:1 as described above. After 24 h of infection, infected macrophages were treated with compound alone and in combinations with the drug for 48 h at 37 °C, 5% CO_2_. The slides were fixed with methanol and stained with 5% Giemsa’s stain solution. Intracellular amastigotes of each treated group were counted and calculated for percentage of growth inhibition compared to untreated control. Results are expressed as mean 95% confidence interval of three experiments.

### 4.9. Analysis of Interaction

Interactions between compounds and the drug were determined based on the combined and single antileishmanial activities. The nature of drug interactions was assessed using the Chou-Talalay combination index method [44]. Each of the combinations combined with non-constant ratio was analyzed using CompuSyn software (ComboSyn Inc., Paramus, NJ, USA). CI values were obtained from non-linear regression results. The equation used was as follows:

$$\mathrm{CI}\;=\;\frac{C_{A,X}}{{\mathrm{IC}}_{x,A}}\;+\;\frac{C_{B,X}}{{\mathrm{IC}}_{x,B}}$$

*C_A,X_* and *C_B,X_* are the concentrations of drug A or drug B in the combination to produce X effect, respectively; IC*_x,A_* and IC*_x,B_* are the concentrations of single drug A or drug B to produce the same effect, respectively. CI value results provided a quantitative determination for strong-to-very-strong synergism (CI < 0.3), synergism (CI = 0.3–0.7), slight-to-moderate synergism (CI = 0.7–0.9), nearly additive (CI = 0.9–1.1), slight-to-moderate antagonism (CI = 1.1–1.45), antagonism (CI = 1.45–3.3) and strong-to-very-strong antagonism (CI > 3.3). The DRI was also calculated, representing the fold of dose reduction that is produced in combination for a given degree of effect, as compared with the dose of each drug alone. Isobolograms were analyzed by using CompuSyn software (ComboSyn Inc., Paramus, NJ, USA) to illustrate a graphical presentation of two drug interactions. A point below, on, or above the line of additivity indicates synergy, additivity, or antagonism, respectively.

### 4.10. Statistical Analysis

Statistical analysis was performed using Excel (Microsoft, Microsoft Corporation, Redmond, WA, USA), CompuSyn software (ComboSyn Inc., Paramus, NJ, USA), and Graphpad software (Graphpad Solfware Inc., San Diego, CA, USA). Mean 95% confidence interval was calculated from triplicate experiments. The statistical differences between groups were evaluated using one-way analysis of variance (ANOVA), followed by the Bonferroni’s multiple comparison tests (using GraphPad prism 6 software (Graphpad Solfware Inc., San Diego, CA, USA)). Differences were considered significant when *p* values were ≤ 0.05.

## Figures and Tables

**Table 1:** Effects of AmB, allicin, and their combinations on intracellular amastigotes of *L. martiniquensis* in peritoneal exudate macrophages (PEMs).

Drug Combination Non-Constant Ratio (μg/mL) ^1^		% Growth Inhibition ^2^	CI ^3^	Interaction	Dose Reduction Index (DRI) ^4^	
AmB	Allicin				AmB	Allicin
0	0	0				
0.0025		25.98 (22.7–29.3)				
0.005		37.79 (32–43.6)				
0.01		52.76 (51.3–54.2)				
	0.16	32.28 (28.1–36.5)				
	0.32	41.73 (39.3–44.1)				
	0.64	51.18 (49.5–52.9)				
0.0025	0.16	54.88 (50.6–59.2) ***	0.63	Synergism	3.02	3.39
0.0025	0.32	66.14 (62–70.3) ***	0.58	Synergism	4.29	2.90
0.0025	0.64	58.27 (56.6–60) ***	1.31	Moderate antagonism	3.34	0.99
0.005	0.16	64.56 (61.1–68.1) ***	0.68	Synergism	2.04	5.36
0.005	0.32	70.63 (65.9–75.4) ***	0.67	Synergism	2.50	3.67
0.005	0.64	60.87 (55–66.8) **	1.44	Moderate antagonism	1.81	1.12
0.01	0.16	70.07 (67.6–72.6) **	0.96	Nearly additive	1.23	7.12
0.01	0.32	66.93 (61.8–72) *	1.24	Moderate antagonism	1.10	3.02
0.01	0.64	69.92 (65.5–74.3) **	1.39	Moderate antagonism	1.22	1.77

^1^ Concentration (μg/mL) of AmB combined with allicin. ^2^ % Growth inhibition (mean 95% confidence interval) obtained from effect of AmB allicin alone, and their combinations. ^3^ CI (Combination index values analyzed by CompuSyn software) classified as strong-to-very-strong synergism (CI < 0.3), synergism (CI = 0.3–0.7), slight-to-moderate synergism (CI = 0.7–0.9), nearly additive (CI = 0.9–1.1), slight-to-moderate antagonism (CI = 1.1–1.45), antagonism (CI = 1.45–3.3), and strong-to-very-strong antagonism (CI > 3.3). ^4^ Dose reduction index (DRI) represents the fold of dose reduction allowed in a combination for a given degree of effect as compared with the dose of each drug or compound alone. Statistical differences between the effects of AmB alone and the combination of AmB plus allicin are indicated as follows: * *p* ≤ 0.01; ** *p* ≤ 0.001; *** *p* ≤ 0.0001.

**Table 2:** Effects of AmB, andrographolide, and their combinations on intracellular amastigotes of *L. martiniquensis* in PEMs.

Drug Combination Non-Constant Ratio (μg/mL) ^1^		% Growth Inhibition ^2^	CI ^3^	Interaction	Dose Reduction Index (DRI) ^4^	
AmB	Andrographolide				AmB	Andrographolide
0	0	0				
0.0025		24.5 (21–28)				
0.005		38.4 (32.3–44.5)				
0.01		51.72 (48.8–54.7)				
	0.08	4.72 (2.68–6.76)				
	0.16	12.6 (9.88–15.3)				
	0.32	44.88 (38.7–51.1)				
	0.64	53.78 (50.7–56.8)				
0.0025	0.08	31.5 (27.8–35.2)	0.93	Nearly additive	1.51	3.79
0.0025	0.16	37.8 (33.7–41.9) **	0.98	Nearly additive	1.87	2.25
0.0025	0.32	51.18 (47.9–54.5) ****	0.98	Nearly additive	2.87	1.58
0.0025	0.64	59.05 (55.5–62.6) ****	1.31	Moderate antagonism	3.67	0.96
0.005	0.08	48.81 (43.8–53.8) *	0.92	Nearly additive	1.33	5.95
0.005	0.16	50.63 (44.8–56.5) *	1.03	Nearly additive	1.41	3.11
0.005	0.32	62.44 (60.3–64.6) ***	0.96	Nearly additive	2.05	2.10
0.005	0.64	51.18 (49–53.3) *	1.97	Antagonism	1.43	0.79
0.01	0.08	40.16 (37.1–43.2) **	2.18	Antagonism	0.51	4.79
0.01	0.16	48.56 (46.1–51.1)	1.85	Antagonism	0.66	2.96
0.01	0.32	37.20 (30.8–43.7) ***	3.08	Antagonism	0.46	1.11
0.01	0.64	44.72 (39.9–49.6) *	3.19	Antagonism	0.58	0.67

^1^ Concentration (μg/mL) of AmB combined with andrographolide. ^2^ % Growth inhibition (mean 95% confidence interval) obtained from effect of AmB, andrographolide alone, and their combinations. ^3^ CI (Combination index values analyzed by CompuSyn software) classified as strong to very strong synergism (CI < 0.3), synergism (CI = 0.3–0.7), slight to moderate synergism (CI = 0.7–0.9), nearly additive (CI = 0.9–1.1), slight to moderate antagonism (CI = 1.1–1.45), antagonism (CI = 1.45–3.3), and strong to very strong antagonism (CI > 3.3). ^4^ Dose reduction index (DRI) represents the fold of dose reduction allowed in a combination for a given degree of effect as compared with the dose of each drug or compound alone. Statistical differences between the effects of AmB alone and the combination of AmB plus andrographolide are indicated as follows: * *p* ≤ 0.05; ** *p* ≤ 0.01; *** *p* ≤ 0.001; **** *p* ≤ 0.0001.
